# Spray Drying Is a Viable Technology for the Preservation of Recombinant Proteins in Microalgae

**DOI:** 10.3390/microorganisms11020512

**Published:** 2023-02-17

**Authors:** Anaëlle Vilatte, Xenia Spencer-Milnes, Harry Oliver Jackson, Saul Purton, Brenda Parker

**Affiliations:** 1 Department of Biochemical Engineering, University College London, Gower Street, London WC1E 6BT, UK; 2 Algal Research Group, Institute of Structural and Molecular Biology, University College London, Gower Street, London WC1E 6BT, UK

**Keywords:** *Chlamydomonas reinhardtii*, microalgae, oral vaccination, recombinant protein, spray drying, scale-up, techno-economic analysis

## Abstract

Microalgae are promising host organisms for the production of encapsulated recombinant proteins such as vaccines. However, bottlenecks in bioprocess development, such as the drying stage, need to be addressed to ensure feasibility at scale. In this study, we investigated the potential of spray drying to produce a recombinant vaccine in microalgae. A transformant line of *Chlamydomonas reinhardtii* carrying a subunit vaccine against salmonid alphavirus was created via chloroplast engineering. The integrity of the recombinant protein after spray drying and its stability after 27 months storage at –80 °C, +4 °C and room temperature were assessed by immunoblotting. The protein withstood spray drying without significant losses. Long-term storage at +4 °C and room temperature resulted in 50% and 92% degradation, respectively. Optimizing spray drying and storage conditions should minimize degradation and favour short-term storage at positive temperatures. Using data on yield and productivity, the economics of spray drying- and freeze drying-based bioprocesses were compared. The drying stage corresponded to 41% of the total production cost. Process optimization, genetic engineering and new market strategies were identified as potential targets for cost reduction. Overall, this study successfully demonstrates the suitability of spray drying as a process option for recombinant protein production in microalgae at the industrial scale.

## 1. Introduction

To meet global food demand, the aquaculture industry had an average growth rate of 5.3% per year between 2001 and 2018 [1]. Of the 114.5 million tonnes of live weight produced in 2018, finfish accounted for more than 47%. The intensification of fish farming raises new challenges in disease management and the delivery of protection at scale. Of particular concern are salmonid alphaviruses (SAVs), which are serious pathogens affecting farmed Atlantic salmon and rainbow trout in Europe [2]. The resulting pancreas (PD) and sleeping diseases can be associated with high mortality rates, e.g., up to 63% [3] in the case of PD, and severe economic losses. Vaccination is an effective strategy to control SAV transmission in fish farming. Conventional vaccination methods involve the intraperitoneal injection [4] of anaesthetized fish with a commercial vaccine such as the multivalent vaccine AquaVac^®^ PD3, which contains inactivated SAV [2]. 

Vaccination by injection can be expensive, labour-intensive and stressful to the fish. An attractive alternative is to administer the vaccine via an oral route [4]. Typically, the production of edible vaccines would involve the microencapsulation of a recombinant subunit antigen and formulation into a feed or supplement [4]. There have been a number of potential oral vaccine candidates for veterinary applications expressed in plants (e.g., lettuce [5]), yeasts [6], and bacterial hosts such as *Bacillus subtilis* [7]. As natural and beneficial components of the aquaculture diet, whole cell microalgae are highly promising systems for such applications [8]. The advantages of microalgae as a production platform for therapeutic proteins have been extensively reviewed elsewhere [9]. Microalgae are photosynthetic microorganisms which can be cost-effectively grown in simple media, with water and sunlight. They can synthesize and correctly assemble a wide range of complex therapeutic proteins [9] such as monoclonal antibodies (mAbs) against the glycoprotein D of the herpes complex virus [10]. In addition, microalgae can be grown at large scale in contained and controlled environments, allowing the implementation of good manufacturing practices (GMP) [9].

The unicellular microalga *Chlamydomonas reinhardtii* is a model organism with well-established genetic engineering tools for both chloroplast and nuclear transformation [11,12]. With its generally regarded as safe (GRAS) status, *C. reinhardtii* represents a promising platform for the production and delivery of edible vaccines [13]. Kiataramgul et al. reported the successful vaccination of shrimp against white spot disease, with a survival rate of 87%, using a transgenic line of *C. reinhardtii* expressing the VP28 viral envelope protein from white spot syndrome virus [14].

Several challenges in process development exist to make microalgae-based oral vaccination feasible and cost-effective at the industrial scale. A crucial step in the manufacturing process is the drying stage, where the dehydration of the algal cells is used both to kill the transgenic algae and to provide a natural method for the protective bioencapsulation of the vaccine [15]. Among the available drying technologies, freeze drying is a gentle technique which is conventionally used at the laboratory scale. Despite its efficiency, freeze drying is considered an expensive technology owing to its high energy requirements, potentially limiting its use at a larger scale of production [16,17]. Several studies have already demonstrated the potential of spray drying to preserve natural compounds, such as β-carotene [18], lipids [19,20], fatty acids [19,20], carbohydrates [19] and proteins, [19] in various microalgal species. However, to our knowledge, no study has investigated the potential of spray drying to preserve microalgae expressing recombinant proteins and, more specifically, to produce microalgae-based edible vaccines for aquaculture applications. For spray drying to be a viable option, the tolerance of the protein to elevated temperatures in the process must be established. In addition, the effect of formulation should be investigated: the microstructure of spray-dried powder has a high surface area [21], which may impact the protein stability. 

The aim of this study was to investigate the suitability of spray drying for the manufacture of microalgae-based edible vaccines at the industrial scale. For this purpose, we created a transgenic line of *C. reinhardtii* expressing a SAV vaccine. The vaccine integrity after spray drying and its stability over time were evaluated. A techno-economic analysis (TEA) was performed using pilot data to forecast the process economics of spray drying compared to freeze drying in a scale-up scenario. 

## 2. Materials and Methods

### 2.1. Algal Strains and Lab-Scale Cultivation

The *C. reinhardtii* strain used as the transformation recipient in this study was the photosynthetic mutant TN72 (CC-5168: *cw15*, *psbH*::*aadA*, mt+), which was previously described in Wannathong et al. [22]. TN72, together with the transformant lines (TN72:E2-ecto) and control lines (TN72:empty and TN72:ptxD), were cultured in tris-acetate-phosphate (TAP) medium [23], which was modified as follows: (NH_4_Cl: 0.400 g/L, K_2_HPO_4_: 0.113 g/L, KH_2_PO_4_: 0.048 g/L, MgSO_4_.7H_2_O: 0.100 g/L, CaCl_2_.2H_2_O: 0.050 g/L, Trizma^®^ base: 2.42 g/L, glacial acetic acid: 1.13 g/L) with a revised trace element recipe (Na_2_EDTA.2H_2_O: 21.5 mg/L, ZnSO_4_.7H_2_O: 0.720 mg/L, MnCl_2_.4H_2_O: 1.19 mg/L, CuCl_2_.2H_2_O: 0.340 mg/L, (NH_4_)_6_Mo_7_O_24_.4H_2_O: 0.035 mg/L, FeCl_3_.6H_2_O: 5.40 mg/L, Na_2_SeO_3_: 0.017 mg/L, Na_2_CO_3_: 2.32 mg/L) [24]. The strains were maintained at 18 °C on 2% TAP agar plates, with TN72 kept under dim light conditions (5–10 µmol.m^−2^.s^−1^) and transformants under moderate light (50–100 µmol.m^−2^.s^−1^) provided by fluorescent tubes. Liquid cultures were cultivated in Erlenmeyer flasks in an illuminated shaking incubator (Innova 4340, New Brunswick Scientific, USA) at 25 °C, 120 rpm, and under constant illumination provided by fluorescent tubes with an average light intensity of 100 µmol.m^−2^.s^−1^. Cultures were routinely tested for contamination on 2% Lysogeny agar (LA) plates incubated in the dark at 37 °C for 2–3 days.

### 2.2. Design and Construction of the SAV Vaccine Transformation Plasmid

**Figure 1:**
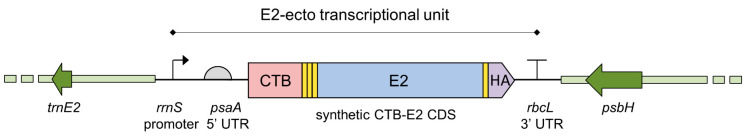
Illustration of the E2-ecto transgene inserted in the *C. reinhardtii* plastome. The four level 0 parts (promoter, 5′UTR, CDS and 3′UTR) were assembled to form the level 1 transcription unit, which was targeted to a neutral site between *psbH* and *trnE2* in the plastome of the *psbH*-deletion mutant TN72, with transformants selected based on restoration of PsbH function, as described by Wannathong et al. [22]. The coding sequence encodes a 511-residue chimeric protein comprising the cholera toxin beta (CTB) subunit, three copies of a GGGGS linker, the SAV E2 ectodomain (E2), a single copy of GGGGS and the HA epitope tag.

The sequence of the SAV vaccine was based on the E2 external glycoprotein domain of the structural protein sequence derived from the genome of a Norwegian isolate of the SAV3 subtype (SAV3-4-SF/10; Genbank Accession number KC122923) [25]. The protein sequence was designed as follows: residues 353 to 730 of the structural polyprotein were fused to the C terminus of the Cholera toxin B subunit sequence [26] via a (GGGGS)x3 flexible linker, and an HA tag (YPYDVPDYA) was added to the E2 C-terminus via a single GGGGS linker. The chimeric protein was termed E2-ecto, and the sequence was back-translated using the codon preference table for the *C. reinhardtii* chloroplast to synthesize a level 0 coding sequence (CDS) part for start–stop assembly [27], a variation of the Modular Cloning (MoClo) assembly system [28]. Full sequence details are given in supplementary Figure S1. The CDS part was then fused to the *C. reinhardtii rrnS* promoter, *psaA* 5′ untranslated region (5′UTR) and *rbcL* 3′UTR using start–stop assembly to create a level 1 transcriptional unit, which was then assembled with left and right homology arms derived from the chloroplast genome, in order to create a level 2 plasmid termed pE2-Ecto that would target the transcriptional unit into the genome, downstream of *psbH* (see Figure 1 and Figure S1).

### 2.3. Transformation of the C. Reinhardtii Chloroplast

Chloroplast transformation of strain TN72 was performed using the vortex method, in which a cell suspension is agitated in the presence of the plasmid and glass beads, followed by plating on high-salt minimal (HSM) medium to select for the restoration of photosynthetic function [22]. Plates were incubated at 25 °C under 50 µmol.m^−2^.s^−1^ white light for 2–3 weeks until colonies appeared. To achieve homoplasmy, transformant lines were restreaked to single colonies two times under selection on HSM plates. Integration of the transgene and homoplasmy were confirmed by a PCR analysis of genomic DNA extracted from a single colony [22].

### 2.4. Cultivation in Single-Use ‘Hanging-Bag’ Photobioreactors

Pilot-scale cultures were carried out in three identical single-use ‘hanging-bag’ (HB) photobioreactors (PBR) [29]. Each PBR was made from heat-sealed polythene layflat tubing (1000-gauge, UK packing, London, UK) with a width of 10.2 cm and a final working volume of 5 L. A late logarithmic-phase culture was used to inoculate the HBs to an initial optical density (OD) reading of 0.015 at 750 nm. The cells were grown in a temperature-controlled room at 24.5 °C under constant illumination provided by three light-emitting diode (LED) panels with an average light intensity of 140 µmol.m^−2^.s^−1^. The cultures were aerated and recirculated using a constant air flowrate of 1 L/min. No carbon dioxide was supplemented. The growth was monitored by off-line OD_750_ readings using semi-micro acrylic cuvettes with a path length of 10 mm and fresh TAP medium as blank.

The cultures were harvested after 3 days upon reaching the end of the logarithmic phase. Before harvesting, each HB was sampled for dry cell weight (DCW) measurements and immunoblotting (biological triplicates). The pellets for DCW were obtained from 50 mL samples after centrifugation (5000 g, 10 min, 12 °C) and stored at –20 °C for future freeze drying. For immunoblotting, 10 mL samples were centrifuged as previously described, snap-frozen in liquid nitrogen and stored at –80 °C for later analyses.

### 2.5. Harvesting by Centrifugation

The cultures were harvested using a tubular bowl centrifuge (CARR Powerfuge P6 Pilot Separation System, Maryland, USA) with manual discharge, operating at 20,000 g at the bowl wall and with a flowrate of 1 L/min from full speed. The temperature was maintained at (22.5 ± 0.5) °C throughout the operation. The feed had a biomass density of (0.57 ± 0.06) g/L DCW and was composed of the three HB cultures pooled together. The cell pellet was manually recovered using a solution of phosphate-buffered saline (PBS) at a pH of 7.4. The slurry was further concentrated for the subsequent drying stage with a final bench-scale centrifugation step (5500 g, 10 min, 12 °C). Samples were taken for DCW measurement and immunoblotting (technical triplicates). In both cases, 1 mL samples were centrifuged for 3 min at 21,100 g. The obtained cell pellets were either directly stored at –20 °C for future freeze drying (DCW) or snap-frozen in liquid nitrogen and stored at –80 °C for later analyses (immunoblotting). After sampling, 55 mL of 9% *w*/*v* slurry ([91 ± 1.6] g/L DCW) in PBS was obtained for the subsequent drying steps. The slurry was stored at +4 °C for 1–2 h during the set-up of the dryers. An amount of 50 mL was used for spray drying while the remaining 5 mL was freeze dried.

### 2.6. Spray Drying

Spray drying was carried out using a laboratory-scale Mini Spray Dryer B-290 (BUCHI, Newmarket, UK) operating in co-current operation. The spray drier was equipped with a two-fluid nozzle using air as the drying medium. The following operating conditions were used: air inlet temperature 120 °C, feed pump rate 3 mL/min (10%), air flow 414 L/min (rotameter height 35 mm) and aspiration 100%. The average outlet temperature was equal to (81.5 ± 1.6) °C and reached a maximum value of 86 °C towards the end of operation. The slurry was kept homogeneous by constant and gentle stirring throughout spray drying. An amount of 3.7 g of spray-dried (SD) powder was recovered. The SD powder was sampled for moisture content analysis and immunoblotting (technical triplicates). One-gram samples were frozen at –20 °C for future freeze drying (moisture content analysis), while 40 mg samples were snap-frozen in liquid nitrogen and stored at –80 °C (immunoblotting).

### 2.7. Freeze Drying

The 5 mL 9% *w*/*v* slurry was centrifuged at 5500 g for 10 min at 12 °C. The obtained cell pellet was snap-frozen in liquid nitrogen and freeze dried for 84 h using a bench-top freeze dryer (Modulyo, Edwards, UK) under vacuum, alongside the samples for the DCW and moisture content analyses. An amount of 0.46 g of freeze-dried (FD) powder was obtained. Again, 40 mg samples were snap-frozen in liquid nitrogen and stored at −80 °C for immunoblotting (technical triplicates).

### 2.8. SDS-PAGE and Immunoblotting

#### 2.8.1. Protein Ladder, Controls and Sample Preparation

The cell pellets, FD and SD powder samples were resuspended in a solution containing 0.8 M of Tris (Trizma^®^ base) and 0.2 M of sorbitol (pH 8.3) to reach an equal biomass density of 2.2 mg/L. A total of 10% *v*/*v* of sodium dodecyl sulphate (SDS) at 10% *w*/*v* and 1% *v*/*v* of β-mercaptoethanol was added to the homogeneous samples. The resulting solutions were then vortexed for 10 s, heated at 100 °C for 5 min using a heat block, cooled on ice for 5 min, vortexed for another 10 s and centrifuged at 21,100 g for 2 min. An amount of 15 µL of each sample was used for the protein gel analysis, which normalized the quantity of biomass loaded per well at 30 µg.

TN72:ptxD, a TN72 transformant strain expressing a 37 kDa HA-tagged PtxD protein, was used as a positive control for the immunoblot [30]. A TN72 strain (TN72:empty) transformed using an empty pSRSapI vector was used as a negative control [22]. The controls were prepared for loading in a similar way to the samples but without normalization.

A broad range (10–250 kDa) colour pre-stained protein standard (NEB#P7719, New England Biolabs Inc.) was used as the protein ladder. An amount of 5 µL was loaded per well.

#### 2.8.2. Electrophoresis, Electroblotting and Antibody incubations

12% SDS-polyacrylamide gels were prepared using a Mini-PROTEAN Tetra cell system (Bio-Rad, Hercules, CA, USA). The manufacturer’s protocol was modified to optimise the visualisation of the studied protein recovered from microalgal cells. Briefly, the electrophoresis was run at 100 V for approximately 2 h. Proteins were transferred to Hybond-ECL nitrocellulose membranes (GE Healthcar, Chicago, IL, USA) at 20 V for 30 min using a Trans-Blot SD semi-dry electrophoretic transfer cell (Bio-Rad). After electroblotting, the membranes were allowed to dry for 20 min before being rehydrated in 1× tris-buffered saline (TBS) for 2 min. Membranes were blocked in a solution of TBS and 0.5% skimmed milk for 1 h at room temperature before being washed in TBS with 0.1% Tween^®^ 20 detergent (TBST) for 15 min. Primary antibody incubation was carried out overnight at +4 °C using rabbit α-HA antibody (Sigma-Aldrich (St. Louis, MO, USA) product H6908, 1:2000 dilution in TBST and 0.5% skimmed milk). The membranes were then washed in TBST for 15 min and 3 × 5 min. Secondary antibody incubation was carried out in the dark for 1 h at room temperature using goat α-rabbit IgG (Thermo Scientific product 35571, Dylight^TM^ 800 4X PEG, 1:25,000 dilution in TBST and 0.5% skimmed milk). In the dark, the membranes were washed in TBST for 15 min and 3 × 5 min, and in TBS for 3 × 5 min. All the blocking, incubation and washing steps were carried out with gentle shaking.

#### 2.8.3. Visualisation and Densitometry Analysis

The membranes were visualised using the Odyssey^®^ Infrared Imaging system (Li-COR Biosciences). For relative quantification, a densitometry analysis was performed using the software LICOR Image Studio Lite (version 5.2.5). Background noise correction was applied using the following settings: median background method with top/bottom borders only to prevent signal overlaps.

### 2.9. Statistical Analysis

Independent two-tailed t-tests were performed to test for equal means between unpaired groups: FD vs. SD samples. The variances were assumed to be equal. Paired difference two-tailed t-tests were used to test for equal means between related groups: culture vs slurry samples, slurry vs. FD or SD samples, and SD samples from different storage conditions. A level of significance α = 0.05 was selected.

### 2.10. Model Construction and Techno-Economic Analysis

#### 2.10.1. Background and Definition of the Case Study

The production of an oral vaccine against SAV in a transformant line of *C. reinhardtii* was used as a case study. The parameters defining the framework of this techno-economic analysis (TEA) are summarised in Table 1.

**Figure 2:**
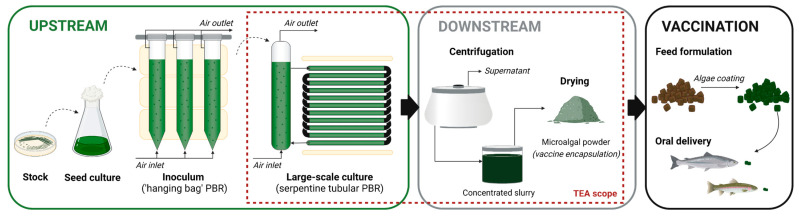
Process steps for the large-scale production of a microalgae-based edible vaccine against SAV, from the preparation of the inoculum for large-scale cultivation in photobioreactors (PBR) to the oral delivery of the vaccine. The scope of the present techno-economic analysis (TEA) is delimited by the red dotted-line rectangle. (Figure created with BioRender.com.)

The main stages required to produce microalgae-based edible vaccines are outlined in Figure 2. In this study, only the process steps from the inoculation of the large-scale PBR to the obtention of the microalgal powder encapsulating the SAV vaccine were considered. The preparation of the inoculum, the sterilisation process and the final vaccine formulation in the feed were not included.

#### 2.10.2. Mass Balances, Bioprocess Design and Operating Strategies

**Figure A1:**
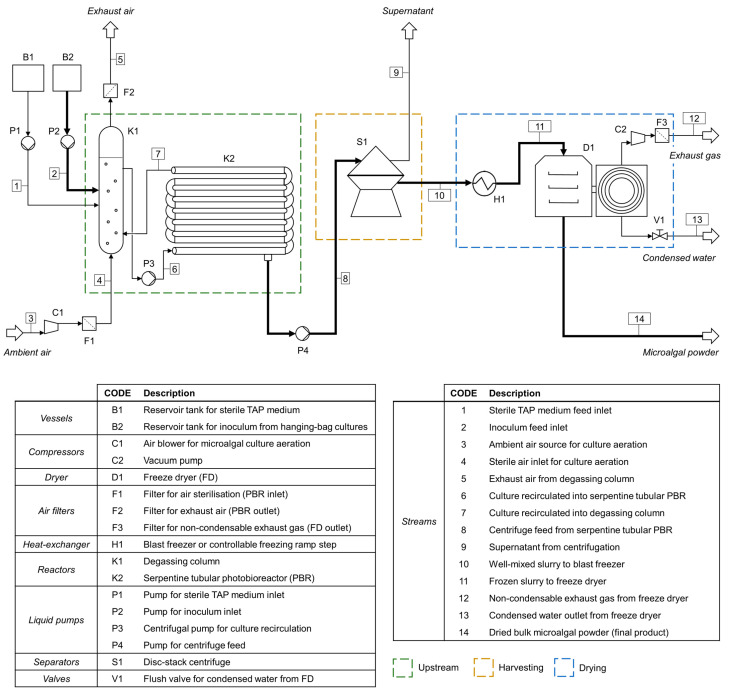
Process flow diagram of the production of a microalgae-based edible vaccine with a freeze-drying strategy.

**Figure A2:**
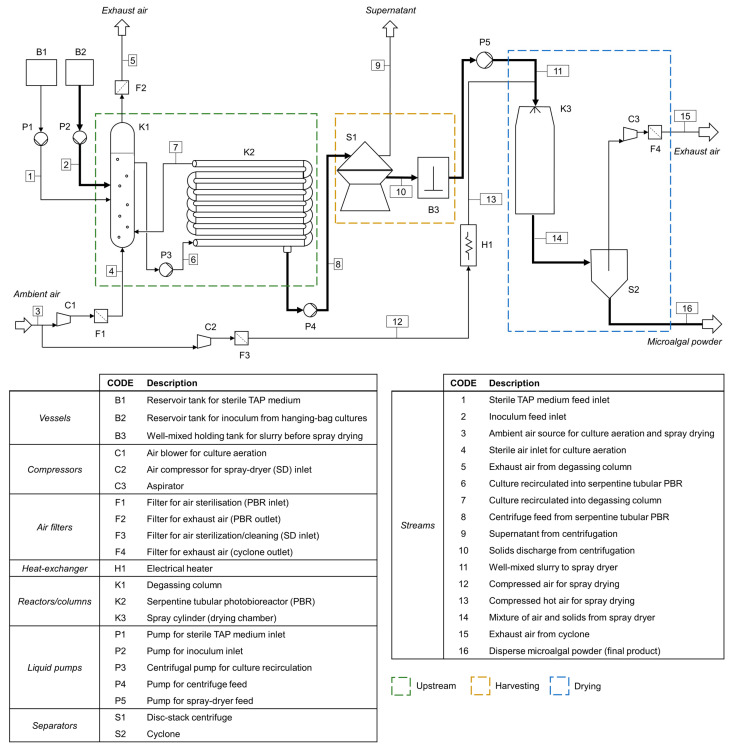
Process flow diagram of the production of a microalgae-based edible vaccine with a spray-drying strategy.

The design of the bioprocess includes a large-scale cultivation system (upstream), a centrifugation step (harvesting) and a drying stage where two technologies are compared: freeze drying vs spray drying. The corresponding process-flow diagrams (PFD) are provided in Appendix A (Figure A1 and Figure A2). The input and output parameters were derived from preliminary experimental data, mass balances and the literature.

In this case study, to account for process losses, the spray-drying strategy was required to process 11% more biomass volume than the freeze-drying strategy to achieve the same annual production target. This directly resulted from the difference in dryer efficiency. For ease of modelling, similar designs and sizing were used for the main equipment of the cultivation and centrifugation steps in both strategies, so there was no change in CAPEX upstream of the drying step. The minimum capacity was based on the requirements of the spray-drying strategy, with an additional 10% oversizing used as a safety margin.

i. Upstream: large-scale cultivation in a serpentine tubular photobioreactor

The large-scale culture of *C. reinhardtii* expressing the SAV vaccine was assumed to be carried out in TAP medium. We considered that the inoculum and sterile growth medium required for the inoculation of the PBR were available and stored in sterile plastic reservoir tanks. A serpentine tubular PBR design was selected to minimise the risks of contamination and ensure the containment of genetically modified organisms (GMO) at scale. The modelled PBR was equipped with a LED light array, a centrifugal pump for culture recirculation, an air blower for culture aeration and a degassing column. We made the assumption that the culture was illuminated by a 12:12 h cycle of artificial and natural lights.

We considered the production of 487 m^3^/year of microalgal culture with the freeze-drying strategy and 541 m^3^/year with the spray-drying strategy, at a biomass density of 0.6 g/L DCW. Evaporation losses were not considered. The annual production target was distributed over 100 batches.

ii. Centrifugation step

In the model, the large-scale culture was harvested using a disc-stack centrifuge to ensure sufficient dewatering efficiency before the subsequent drying stage [33]. A hydro hermetically sealed design was selected to minimize the level of shear stress experienced by the cells in the feed zone [34]. The centrifuge was also assumed to be equipped with a modified discharge mechanism, averting the shear damage described in previous work [35]. A maximum holding time of 2 h/batch was considered to prevent slurry degradation and vaccine losses. Considering the batch size of the spray-drying strategy, the holding time and the 10% safety margin, a minimum capacity of 3 m^3^/h was required. A design with a maximum operating flowrate of 5 m^3^/h was selected to ensure sufficient separation efficiency to reach a degree of clarification of 98%. The centrifugation step allowed the recovery of 29.5 kg of slurry with the freeze-drying strategy and 32.8 kg of slurry with the spray-drying strategy at 10% *w*/*v* (100 g/L DCW).

After centrifugation, a mixing tank equipped with a three-blade marine propeller was considered for the spray-drying strategy. This additional mixing step aimed to keep the slurry homogenized before spray drying.

iii. Freeze-drying strategy

The capacity of the freeze dryer should accommodate the sublimation of 26.6 kg_water_/batch with the production of 2.9 kg_DCW_/batch. As freeze drying is carried out in batch operation, losses were considered negligible. The residual moisture content of the FD powder (reported as <10%) [36,37] was assumed to be negligible as well.

A freeze dryer equipped with bulk drying trays and a controllable freezing ramp was selected. The freeze dryer has a total ice condenser capacity of 50 kg, which should allow approximately 70% of the slurry per batch to be processed. Therefore, two 50 L freeze-dryer units were considered to freeze dry the whole batch at once and prevent slurry degradation and vaccine losses. A maximum drying cycle of 60 h was considered.

iv. Spray-drying strategy

Spray drying was operated continuously with a maximum holding time of 2 h/batch to prevent slurry degradation and vaccine losses. In our pilot-scale experiments, a drying efficiency of 82% was achieved with non-optimal operating conditions. For this case study, a drying efficiency of 90% was considered. The spray dryer design should ensure the evaporation of 29.9 kg_water_/batch with the production of 2.9 kg_DCW_/batch. The residual moisture content of the SD powder (reported as <10%) [19,36,37] was assumed to be negligible. A pilot-scale spray dryer with an evaporating capacity of 15 kg_water_/h was selected.

#### 2.10.3. Economic Model and Key Assumptions

All economic data are expressed in £GBP_2019_ (hereafter, £) using the Chemical Engineering Plant Cost Index (CEPCI) and the Organisation for Economic Co-operation and Development’s (OECD) currency exchange rates [38] as appropriate.

i. Capital expenditures (CAPEX)

A preliminary cost estimate (class 4, ±30% accuracy) [39] was carried out using limited design details and costing data obtained from suppliers. The estimation of the inside battery limits (ISBL) capital investment was based on the total purchased costs of the main equipment items and a Lang factor of 4 (mixed fluids-solids processing plant) [39]. The design of the main equipment items and corresponding costing data are provided in Appendix B (Table A1). The offsite battery limits (OSBL) investment was excluded. Contingency charges were added at 10% of the IBSL [39].

ii. Operating expenditures (OPEX)

The OPEX include variable costs of production (utilities, consumables) and fixed costs of production (labour, maintenance, insurance).

The electricity consumption was calculated considering the energy requirement of the main items and their operating time. Energy requirements were provided by suppliers unless otherwise stated. The price of electricity was set at 0.149 £_2019_/kWh [40] (small industry consuming 20–499 MWh/year, including the Climate Change Levy tax). Water consumption and chemical usage were mainly attributed to the preparation of the culture medium. Auxiliary water usage and additional chemicals used for cleaning, or pH control, were not included in this study. The price of water was set at 1.5 £_2019_/m^3^. The costs associated with air consumption for culture aeration and spray drying were considered negligible.

The plant is expected to operate for 300 days per year allowing additional time for equipment maintenance and contingencies. Labour requirements were estimated based on the workload for the freeze-drying and spray-drying strategies. Supervision costs were estimated at 25% of the total salaries [41]. Direct salary overhead was also included at 50% of the total salaries and supervision costs. The maintenance and insurance costs were estimated at 5% and 1% of the ISBL investment, respectively.

The complete details of the considered variable and fixed production costs are provided in Appendix B (Table A2 and Table A3).

iii. Capital charges (A) and total cost of production (TCOP)

The annual capital charges were based on a loan period of 10 years and an interest rate of 10% [42]. The total cost of production resulted from the addition of the annual capital charges to the OPEX. The TCOP was used to determine the cost of production of the microalgal powder (£_2019_/kg_DCW_) and the SAV vaccine dose (£_2019_/unit).

## 3. Results and Discussion

### 3.1. Investigating the Feasibility of Spray Drying for Downstream Processing

#### 3.1.1. Influence of Spray Drying on Recombinant Protein Integrity

**Figure 3:**
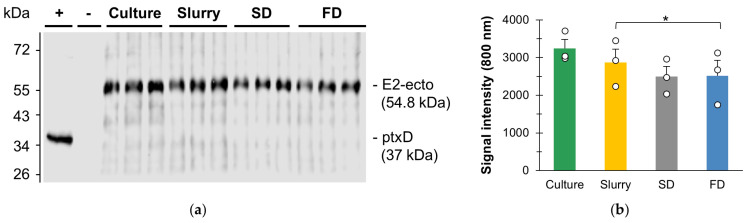
Evaluation of E2-ecto vaccine integrity throughout the manufacturing process, with a focus on the effects of spray drying as compared to freeze drying. (**a**) Immunoblot analysis of E2-ecto vaccine in TN72:E2-ecto cells after cultivation (“culture”, biological triplicates), centrifugation (“slurry”, technical triplicates), spray drying (“SD”, technical triplicates) or freeze drying (“FD”, technical triplicates). Samples were normalized at 30 µg of biomass loaded per well. Positive control (+): TN72:ptxD, negative control (–): TN72:pSRSapI. (**b**) Densitometry analysis of the culture (biological triplicates), slurry, spray-dried (SD) and freeze-dried (FD) samples (technical triplicates) in the 800 nm channel. Data are expressed as the average signal intensity ± one standard error. Individual data points are shown as white circles. Student’s t-tests were applied, with statistically significant differences identified with * (*p*-value < 0.05).

The experimental part of this study aimed to evaluate the feasibility of using spray drying for the generation of dried whole cell powder from a microalga engineered to produce a recombinant protein. Here, an oral vaccine against SAV produced in the chloroplast of a cell-wall-deficient strain of *C. reinhardtii* was used as a case study. Specifically, we investigated whether the heat applied during the spray-drying process would result in partial or complete degradation of the vaccine, and how this would compare to the freeze-drying performance. For this purpose, strain TN72:E2-ecto was grown at pilot scale to produce sufficient biomass for downstream processing (DSP). The vaccine integrity in the microalgal cells was monitored by immunoblotting throughout the process by sampling the cultures upon harvesting, the 9% *w*/*v* slurry, the spray-dried (SD) and freeze-dried (FD) powders. The results are shown in Figure 3a.

The immunoblot revealed a unique band at ~55 kDa in all samples, which corresponds to the predicted size (54.8 kDa) for the CTB-E2-HA chimeric protein referred to as ‘E2-ecto’ (Figure 3a). This first result confirms that the vaccine was present and intact in the SD powder. A densitometry analysis was then performed to assess potential vaccine losses during the manufacturing process and, specifically, to compare the performance of spray drying with freeze drying (Figure 3b). The statistical analysis did not identify significant vaccine losses from centrifugation, with a calculated *p*-value of 0.19 (<0.05). When comparing the signal intensity in the slurry with the SD powder, no definitive conclusion could be drawn regarding the effects of spray drying. The calculated *p*-value = 0.054 was very close to the level of significance α. Spray drying may have caused some levels of vaccine degradation, but the current experimental set up did not identify such an effect. In contrast, differences in signal intensity measured in the slurry and the FD powder were statistically significant, with a calculated *p*-value of 0.039. Before freeze drying, the pellet was snap-frozen in liquid nitrogen to ensure the formation of small ice crystals and minimize cell damage [21]. The apparent vaccine losses here could be due to residual heterogeneities in the rehydration process of the FD samples during sample preparation. This is highlighted by the spread of the data among the technical triplicates, which is characterized by a coefficient of variation of 28%. The FD samples were more challenging to rehydrate than the SD samples, which might be due to the differences in their three-dimensional (3D) structures [21]. Unlike freeze drying, spray drying allows the formation of hollow spherical particles, which enhances the exchange surface area. Finally, there were no statistically significant differences between the signal intensities of the SD and FD powders (*p*-value = 0.96).

Spray drying led to similar results to freeze drying, and the integrity of the SAV vaccine was maintained during the spray-drying process with the operating conditions tested in this study. Zhang et al. also demonstrated that spray drying and freeze drying resulted in similar total lipid, protein, carbohydrate and starch contents, and fatty acid composition in the green microalga *Scenedesmus acuminatus* [19]. Another study using the marine diatom *Phaeodactylum tricornutum* demonstrated that the total lipid and free fatty acid contents were similar in freeze-dried and spray-dried powders [20]. Although Leach et al. successfully recovered more than 90% of β-carotene in spray-dried *Dunaliella salina* [18], several studies reported 25–30% of degradation in total carotenoids or astaxanthin content after spray drying [20,36,37].

Although spray drying showed potential as an alternative to freeze drying, further research needs to be carried out to confirm whether recombinant proteins retained their biological activities and properties of interest in the SD powder. In the case of edible vaccines, the antigenicity of the spray-dried vaccine would need to be tested in fish trials and compared to conventionally used vaccines.

#### 3.1.2. Stability of SAV Vaccine in Spray-Dried Powder over Time

**Figure 4:**
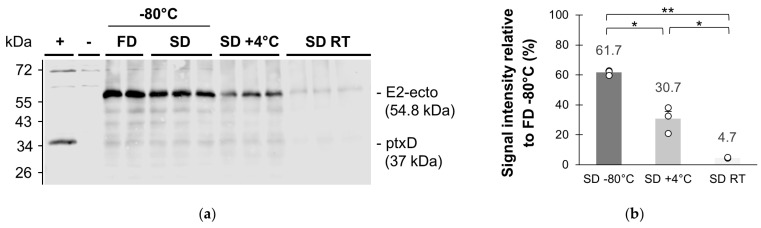
Evaluation of E2-ecto vaccine stability in spray-dried microalgal powder after long-term storage at different temperatures. (**a**) Immunoblot analysis of E2-ecto vaccine in spray-dried (SD) microalgal powder stored at –80 °C, +4 °C and ambient temperature (RT) for 27 months. The freeze-dried (FD) microalgal powder stored at –80 °C in similar conditions was used as reference. Each sample was analysed in technical triplicates, except for the FD powder, for which one of the replicates was lost during storage. Samples were normalized at 30 µg of biomass loaded per well. Positive control (+): TN72:ptxD, negative control (–): TN72:pSRSapI. (**b**) Densitometry analysis in the 800 nm channel of the spray-dried (SD) samples stored at –80 °C, +4 °C and ambient temperature for 27 months. Data are expressed as the average signal intensity relative to the average signal intensity of the FD powder stored at –80 °C ± one standard error. Individual data points are shown as white circles. Student’s t-tests were applied, with statistically significant and highly significant differences identified with * (*p*-value < 0.05) and ** (*p*-value < 0.01), respectively.

In order to predict the shelf life of microalgae-based edible vaccines, the stability of the recombinant protein in the spray-dried powder over time and the impacts of the storage conditions were examined. For this purpose, samples of SD powders were aliquoted in 1.5 mL Eppendorf tubes and stored at –80 °C, +4 °C and ambient temperature, for 27 months before immunoblotting (Figure 4a).

The immunoblot revealed that the vaccine was still present in the spray-dried powder samples stored at –80 °C, +4 °C and ambient temperature after long-term storage. However, the densitometry analysis identified statistically significant differences in signal intensities between the samples, which accounted for different degrees of vaccine degradation during storage (Figure 4b).

The vaccine concentration in the SD powder stored at –80 °C was approximately 38% lower than in the FD powder (*p*-value = 10^−4^ < 0.01). In the freshly processed material, a reduction in signal intensity accounting for vaccine degradation after spray drying was not detected (Figure 3). Therefore, these losses might result from the environmental conditions the powder was subjected to between its collection and the start of the stability study. The SD powder was stored at room temperature for a few days prior to being frozen, which may have initiated vaccine degradation. Leach et al. reported a rapid degradation of β-carotene in the spray-dried powder of *D. salina*, whose content decreased below 10% of the initial carotene level after 7 days of storage at room temperature in the presence of light and oxygen [18]. However, as the same SD powder was aliquoted for the stability study after this short-term storage at room temperature, the initial degradation does not have an impact on the relative comparison of the results obtained at –80 °C, +4 °C and ambient temperature (Figure 4b).

Long-term storage at +4 °C and ambient temperature resulted in 50% (*p*-value = 0.029 < 0.05) and 92% (*p*-value = 3.5 × 10^−4^ < 0.01) vaccine losses in the SD powder, respectively, as compared to long-term storage at –80 °C. Here, the storage temperature and the level of vaccine degradation were positively correlated. Unlike the samples stored at –80 °C and +4 °C, the samples stored at ambient temperature were subjected to an additional source of stress with natural fluctuations in light and temperature. All the studied samples were also exposed to air.

Previous studies reported that the degree of oxidation in SD powder is higher upon air exposure and was positively correlated to the storage time and temperature [20,36,43]. Ryckebosch et al. studied the stability of vacuum-packed and non-vacuum-packed spray-dried *P. tricornutum* after 14 and 35 days of storage at −20 °C, +4 °C and +20 °C [20]. In this short period of time, the study did not find any significant effects of the storage conditions on the total lipid and carotenoid contents. Raposo et al. evaluated the astaxanthin content of spray-dried *Haematococcus pluvialis* after 9 weeks of storage at −21 °C or +21 °C under vacuum, nitrogen or air exposure [43]. Depending on the spray-drying conditions tested, the study reported degradation levels between approximately 50 and 70% after 6 weeks of storage at +21 °C with air exposure. Storage under vacuum or nitrogen atmosphere reduced the degree of degradation down to a minimum of 35%. Ahmed et al. also investigated the effects of different storage conditions on the astaxanthin content of spray-dried *H. pluvialis* after 20 weeks of storage [36]. Degradation levels of approximately 50% and 80% were reported with air exposure at +4 °C and +20 °C, respectively. The study also found that vacuum packing reduced the levels of degradation to approximately 35% and 45% at +4 °C and +20 °C, respectively.

Although vaccine degradation was potentially expected after more than 2 years of storage, these preliminary results show promise for short-term storage at positive temperatures. Future research for aquaculture applications would need to evaluate the stability of recombinant proteins in SD microalgal powder after a few weeks of storage, which was not possible in the scope of this study. The stability of the recombinant proteins in SD powder should also be maximized by considering vacuum packaging or inert atmosphere for storage. Natural fluctuations in temperature and light would need to be minimized using a controlled environment and dark conditions.

In addition to storage, the host biology and the selection of the drying conditions may also have an influence on the stability of the recombinant proteins in the SD powder over time. From a molecular engineering perspective, selecting a strain with an intact cell wall could ensure the presence of an additional protective layer. The cell wall and chloroplast membranes would then act as a natural method of bioencapsulation [15] to minimize vaccine degradation during drying and storage.

The spray-drying conditions used to produce the microalgal powder have also been reported to influence the stability of the component of interest over time [43]. Raposo et al. studied the effects of the inlet and outlet temperatures during spray drying on astaxanthin degradation depending on the storage conditions [43]. At 21 °C under vacuum, the level of degradation was approximately twice as high in the SD powder dried at temperatures of 220/120 °C (inlet/outlet) than 200/80 °C. Therefore, future work should consider the influence of the combination of spray drying and storage conditions to further optimize the stability of recombinant proteins in the SD powder.

### 3.2. Evaluation of Spray-drying Potential at Industrial Scale

Following the experimental study, a techno-economic analysis (TEA) was performed to evaluate the feasibility of manufacturing microalgae-based edible vaccines at scale. Although realistic figures were used, it is important to mention that the proposed flowsheets were not optimized to minimize the costs of production. The main objectives were to identify the current bottlenecks in process development and to investigate how the potential of spray drying could be realised at the industrial scale. As a case study, we considered the production of the SAV vaccine in *C. reinhardtii* with a view to meeting the vaccination demand for Atlantic salmon and rainbow trout farming in the United Kingdom (UK). Process strategies relying on either spray drying or freeze drying were compared.

#### 3.2.1. Base Case Scenario: Economics and Scale of Production

**Figure 5:**
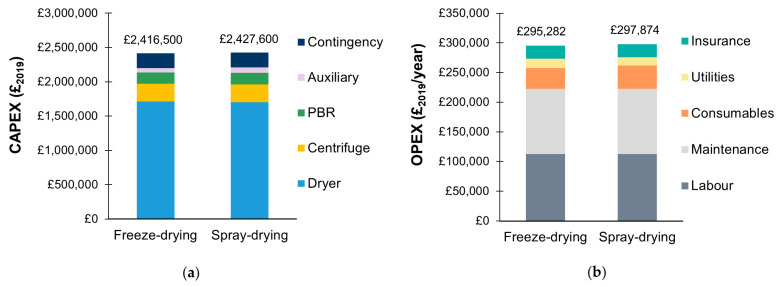
Details of the (**a**) capital expenditures (CAPEX) and (**b**) operating expenditures (OPEX) for the production of a microalgae-based edible vaccine with a freeze-drying or spray-drying strategy. An annual production of 286 kg_DCW_ of microalgae is considered with a vaccine yield of 3.15 g_vaccine_/kg_DCW_ (base case scenario).

Irrespective of the drying technology, the production of 286 kg_DCW_ of microalgae per year was associated with a capital investment (CAPEX) of approximately £2,400,000 and operating costs (OPEX) of £300,000 **(**Figure 5). The CAPEX depreciation over 10 years accounted for 57% of the total cost of production (TCOP). Both strategies were associated with final costs of production of approximately £2400 per kilogram of microalgal powder and £0.008 per unit of vaccine dose. Both the spray dryer and the freeze dryer were the major cost contributors in terms of capital investment: they accounted for more than 70% of the CAPEX. Maintenance and labour were the main contributors to the operating costs, accounting for 38% and 37% of the OPEX, respectively.

The final cost of production of the microalgal powder is very high as compared to other results found in the literature. For transparency, the costs of production mentioned in the following section are reported as they were originally written in the literature. However, these figures are associated with different years of study, currencies and geographic locations. Previous techno-economic analyses reported costs of production of €4.16/kg_DCW_ [44], €12.4/kg_DCW_ [45] and $32.16/kg_DCW_ [16] when considering the obtention of a wet algal paste, and €53.32/kg_DCW_ [46], €69.3/kg_DCW_ [47] and €105.19/kg_DCW_ [46] with an additional drying step. However, these studies benefited from the economy of scale, as they were carried out at a significantly larger scale of production than the one considered in this case study, with productivities ranging from 3.8 [47] to 41 tonnes per year [44]. In these studies, the design of the process had also been optimized to minimize the costs of production, e.g., novel photobioreactor design [48], use of seawater and natural light [16,44,45,46,47].

While the biomass production cost is estimated at approximately €50/kg_DCW_ at the 1000s kg scale [49], biomass costs can be much higher at the 100s kg scale. This scale of production is usually encountered in aquaculture applications where biomass costs can be higher than €500/kg_DCW_ [49]. To our knowledge, only two studies focused on the economics of microalgae production in small-scale facilities for aquaculture applications. In 1992, Coutteau et al. surveyed 50 commercial and experimental aquaculture hatcheries worldwide to investigate the costs of microalgal production [50]. The dry biomass cost ranged from $50 to $400 per kilogram. Recently, Oostlander et al. carried out a techno-economic analysis to investigate the cost of production of microalgal biomass in aquaculture hatcheries, considering an annual production of 125 kg_DCW_ [51]. The culture was carried out continuously using tubular PBR and artificial light. They obtained a final biomass cost of €290/kg_DCW_. It is important to mention that in both studies, the final product considered was the diluted microalgal culture. The studies did not include the costs of harvesting and drying, which here accounted for approximately 6% and 41% of the final biomass cost, respectively, for both the freeze-drying and spray-drying strategies.

Considering increasing the size of the targeted market and, therefore, the scale of production, could alleviate the negative impacts of small production requirements. The latter had already been suggested by Oostlander et al. who highlighted the potential for cost reduction by centralizing microalgae production for aquaculture applications [51].

#### 3.2.2. Influence of the Drying Stage

In the present study, the economic importance of the spray dryer and freeze dryer resulted from the scale of production and the large drying capacity required to evaporate the water content of the 10% *w*/*v* slurry. The required drying capacity directly impacted the size of the instrument and, therefore, the associated capital investment (more than 77% of the total equipment cost). Processing a more concentrated slurry could help to reduce the economic burden associated with the drying stage. Unlike freeze drying, additional research might be needed for spray drying to define the maximum solids content, which can be processed while ensuring the feed can still be pumped and atomized.

Interestingly, Acién et al. found that the PBR represented 48% of the total equipment cost, followed by the freeze dryer at 31.6%, with an annual biomass production of 3.8 tonnes per year [47]. Increasing the biomass production to 200 tonnes per year resulted in the PBR accounting for 94% of the total equipment cost. Vázquez-Romero et al. discussed similar cost distributions when considering an annual biomass production of 27.6 tonnes per year [46]. In their study, the PBR accounted for 66.8% of the major equipment costs, while the freeze dryer only represented 25.9%. These results show that potential bottlenecks in process development are different depending on the scale of production. The drying stage is prominent at the 100s kg scale, while the cultivation system becomes the major cost contributor at the 1000s kg scale. This scale effect is essential to consider when looking at strategies for optimization and cost reduction.

#### 3.2.3. Influence of the Vaccine Yield

In a different scenario, we investigated how potential improvements in productivity would influence the process economics. Specifically, the impact of re-engineering the *C. reinhardtii* strain to maximise the vaccine yield by a three-fold increase was assessed. Here, we considered a strain producing 9.45 g_vaccine_/kg_DCW._ The process designs and scale of production in terms of biomass quantity were kept identical to the base case scenario, and the vaccine surplus was exported outside the UK. The increase in vaccine yield allowed a reduction in the vaccine dose cost of production by 66.7%, reaching a unit cost of £0.0025 (FD) and £0.0026 (SD). Therefore, maximising the vaccine yield through genetic engineering should constitute one of the next research targets to ensure the cost-effectiveness of microalgae-based edible vaccine production at scale.

#### 3.2.4. Additional Considerations on the Selection of the Drying Technology

Beyond the process economics, freeze-drying and spray-drying technologies present different characteristics in terms of design, operations and dried powder properties, which may influence the decision-making process. First, spray drying allows the formation of a fine, dispersible powder composed of hollow spherical particles, while freeze-dried powder is composed of 3D networks [21]. These differences in microstructure should be considered with regard to the final application of the recombinant protein and the following formulation steps. Secondly, spray drying is a continuous technology which allows a higher flexibility in operation than freeze drying. The latter is conventionally operated in batch and requires a longer operating time than spray drying. Koopman et al. thoroughly reviewed associated considerations in terms of operating strategies, maintenance, cleaning and workload [37], which should also be considered and tailored to the studied process. The intensity at which the capital equipment is used by the process will also influence the investment; however, this can be ascertained with greater certainty in discussion with equipment manufacturers. Life cycle assessment can also be applied at the design stage to investigate the sustainability implications of technology selection.

## 4. Conclusions

To our knowledge, this is the first study investigating the suitability of spray drying to produce recombinant proteins in microalgae at the industrial scale. The production of a microalgae-based edible vaccine against SAV for aquaculture applications was used as a case study. We have shown that spray drying was a feasible alternative to freeze drying, preserving the vaccine integrity during operation without significant losses. Further research is still required to confirm whether the recombinant proteins retained their bioactivities in the spray-dried powder. Although vaccine degradation was observed during storage for 27 months at temperatures of +4 °C or above, the obtained results showed promise for short-term storage without the requirement of a cold chain. Similar stability of other recombinant proteins in dried *C. reinhardtii* has been reported by several groups [12]. A further improvement in the protein stability may be achieved by adjusting the storage conditions and protocol for formulation. We have also discussed the importance of spray-drying conditions, which need to be optimized to maximize the process efficiency and the stability of the protein in the spray-dried powder.

The techno-economic analysis highlights that the drying stage is the major cost contributor at the 100s kg production scale. A combination of process optimization, genetic engineering and new market strategies need to be implemented to minimize the cost of production at this scale. The spray-drying strategy is shown to be as competitive as the freeze-drying strategy.

Beyond the process economics, additional considerations, such as the final application of the microalgal powder and the operating strategy considered, may help inform the selection of the most suitable drying technology. At this point, bioprocess design for algal operations is bespoke to the application, but this study successfully demonstrates the potential of spray drying to produce recombinant proteins in microalgae at the industrial scale.

## Figures and Tables

**Table 1:** Details of the case study on the industrial production of a microalgae-based edible vaccine against SAV for aquaculture applications.

Parameters	Details	Reference/Notes
Location	United Kingdom (UK)	Market, process location
Year of study	2019	
Vaccination targets	Atlantic salmon and rainbow trout	82,000,000 fish/year (UK) ^1^
Vaccine dose	10 µg_vaccine_/fish	Industry communication
Microalgal strain	TN72:E2-ecto	
Vaccine yield	3.15 g_vaccine_/kg_DCW_	Experimental results (this study)
Annual production	286 kg_DCW_/year	10% safety margin
Operating time	300 days/year	

^1^ Estimated from UK aquaculture production statistical data from the Food and Agriculture Organization of the United Nations (FAO) [31] and the Marine Scotland Directorate [32]. Average fish weights of 0.5 kg for freshwater rainbow trout and 4 kg for Atlantic salmon were considered to estimate the total number of fish to be vaccinated.

## Data Availability

The data supporting the conclusions of this study are available in this article, or in the cited articles where they were originally reported. Additional data are available on request from the corresponding author.

## Supplementary Materials

The following supporting information can be downloaded at: https://www.mdpi.com/article/10.3390/microorganisms11020512/s1, Figure S1: Sequence details of the E2-ecto transgenes.

## Appendix B. Design Details, Capital Investment and Operating Costs

**Table A1:** Equipment sizing and associated capital costs for the freeze-drying and spray-drying strategies. Values provided in the upstream and harvesting sections are identical for both strategies unless otherwise stated.

PFD Code	Process Step	Equipment	Capacity	Nb Units	Lifespan (Years)	Total Installed Cost ^1^
B1	Inoculation	Plastic reservoir tanks	5.3 ^a^–5.9 ^b^ m^3^	1	10	£21,700 ^a^–£24,100 ^b^
B2			0.08 ^a^–0.09 ^b^ m^3^	1	10	£300 ^a^–£400 ^b^
P1		Pumps	5 m^3^/h	1	20	£20,600
P2			0.1 m^3^/h	1	20	£16,200
C1, F1, K1, F2, P3, K2	Cell culture	Serpentine tubular PBR with LED array	6.01 m^3^	1	10	£165,400
**Upstream sub-total**						**£224,200 ^a^–£226,700 ^b^**
P4, S1	Harvesting	Disc-stack centrifuge	5 m^3^/h	1	50	£260,700
B3		Mixing tank ^b^	0.05 m^3^	1	20	£17,700 ^b^
**Harvesting sub-total**						**£260,700 ^a^–£278,400 ^b^**
H1, D1, C2, F3, V1	Drying	Freeze dryer ^a^	50 kg_ice_	2	20	£1,711,900 ^a^
C2, F3, H1, P5, K3, S2, C3, F4		Spray dryer ^b^	15 kg_water_/h	1	20	£1,701,800 ^b^
**Drying sub-total**						**£1,711,900^a^–£1,701,800 ^b^**

^1^ Total installed cost including a Lang factor of 4 when appropriate. ^a^ Freeze-drying strategy. ^b^ Spray-drying strategy.

**Table A2:** Variable production costs for the freeze-drying and spray-drying strategies. Values provided in the upstream and harvesting sections are identical for both strategies unless otherwise stated.

PFD Code	Process Step/Details	Equipment	Energy	Annual Consumption	Annual Costs
P3	Culture recirculation	PBR ^2^	200 W/m^3^_culture_	5844 ^a^–6493 ^b^ kWh/year	£871 ^a^–£967 ^b^
K2	Culture illumination		4 W/L_PBR_	72,146 kWh/year	£10,750
-	Chemicals (TAP medium) ^1^		-	-	£34,954 ^a^–£38,838 ^b^
-	Water (TAP medium)		-	478 ^a^–531 ^b^ m^3^/year	£717 ^a^–£797 ^b^
P4, S1	Centrifugation	Disc-stack centrifuge ^3^	1.5 kWh/m ^3,4^	730 ^a^–812 ^b^ kWh/year	£109 ^a^–£121 ^b^
H1, D1	Drying	Freeze dryer ^a^	2 kWh/h	24,000 kWh/year	£3576 ^a^
H1, K3		Spray dryer ^b^	50 kWh/h	9969 kWh/year	£1485 ^b^
**Total variable production costs (utilities + consumables)**					**£50,976 ^a^–£52,958 ^b^**

^1^ TAP medium price calculated at 72.9 £_2019_/L based on individual chemical prices. ^2^ The energy consumption of the air blower and the pumps used for inoculation was considered negligible. The heating and cooling requirements were not included. ^3^ The energy consumption of the mixing tank keeping the slurry homogenized during spray drying was considered negligible. ^4^ (0.7–2.4 kWh/m^3^) [52,53]. ^a^ Freeze-drying strategy. ^b^ Spray-drying strategy.

**Table A3:** Fixed production costs for the freeze-drying and spray-drying strategies. Values provided are identical for both strategies unless otherwise stated.

Value	Details	Annual Costs
Labour	1× full-time biochemical engineer	£40,000
	2× part-time (2/5) production operators	£20,000
Supervision, management	25% operating labour [41]	£15,000
Direct salary overhead	50% operating labour + supervision [41]	£37,500
Maintenance	5% ISBL [41]	£109,838 ^a^–£110,346 ^b^
Insurance	1% ISBL [41]	£21,968 ^a^–£22,069 ^b^
**Total fixed production costs (labour + maintenance + insurance)**		**£244,306 ^a^–£244,915 ^b^**

^a^ Freeze-drying strategy. ^b^ Spray-drying strategy.
