# Supplementary material for: Spray Drying Is a Viable Technology for the Preservation of Recombinant Proteins in Microalgae
Source: Microorganisms. 2023 Feb 17;11(2):512. doi: 10.3390/microorganisms11020512 (PMC9967251; doi:10.3390/microorganisms11020512)
Supplement: Supplementary file 1 [file microorganisms-11-00512-s001.zip › microorganisms-2178438-supplementary.pdf]

Supplementary Figure S1: Sequence details of the E2-ecto transgenes

>DNA sequence of E2-ecto transcriptional unit

aaaacaattattattttactgcggagcagcttgttattgaaattttattaaaaaaaaaataa
aaatttgacaaaaaaaaataaaaaagttaaattaaaaacactgggaatgttctacatcataa
aaatcaaaagggtttaaaatcccgacaaaatttaaactttaaagagtatgatgtaaaaaaaa
ctatttgtctaatttaataaccatgcatttttttatgaacacataataattaaaagcgttgct
aatggtgtaaataatgtatttattaaattaaataattgttattataaggagaaatccATGAC
ACCACAAAACATTACTGACTTATGCGCTGAATACCACAACACTCAAATTTACACTTTAAATG
ACAAAATTTTCTCTTATACAGAATCTTTAGCTGGTAAACGTGAAATGGCAATCATTACTTTT
AAAAACGGTGCTATTTTCCAAGTTGAAGTACCAGGTTCACAACACATTGATTCACAAAAAAA
AGCTATCGAACGTATGAAAGATACTTTACGTATTGCTTACTTAACTGAAGCTAAAGTTGAAA
AATTATGTGTATGGAATAACAAAACTCCACACGCAATTGCTGCTATTTCTATGGCTAATGGT
GGTGGTGGTAGTGGCGGTGGTGGTAGCGGTGGTGGTGGTTCAGCAGTAAGTGCTTCTCCAGC
GGCTGTTTATGATACTCAAATTTTAGCTGCTCACGCAGCTGCATCTCCATACCGTGCTTACT
GTCCTGATTGTGACGGTACTGCATGTATTTCTCCAATTGCAATTGATGAAGTTGTATCATCA
GGTTCTGACCACGTATTACGTATTCGTGTAGGTTCACAATCTGGTGTAACTGCTAAAGGTGG
TGCTGCTGGTGAAACTTCATTACGTTACTTAGGTCGTGACGGTAAAGTTCACGCTGCTGATA
ACACTCGTTTAGTAGTTCGTACAACTGCTAAATGTGACGTTTTACAAGCAACAGGTCACTAC
ATTTTAGCTAGTTGTCCAGAAGGTCAATCAATTACAGTAGCTGCTACATTAGATGGTACTCG
TCACCAATGTACTACAGTATTCGAACACCAAGTAACTGAAAAATTTACACGTGAACGTTCTA
AAGGTCACCACTTAAGTGATTTAACTAAAAAATGTACACGTTTCAGTACAACACCAAAAAAA
TCAGCTCCATACTTAGTAGATGTTTACGATGCTTTACCAATTTCTGTAGAAATTTCTACAGT
AGTTACTTGTAACGATAACCAATGTACTGTACGTGTTTCTCCAGGTACAACTGTAAAATTTG
ATAAAAAATGTAAAAGTGCTGCTCAAGCTACAGTTACTTTCACATCTGATTCTCAAACATTT
ACTTGTGAAGAACCAGTTTTAACTGCTGCTAGTATTACTCAAGGTAAACCACACTTACGTAG
CTCAATGTTACCATCAGGTGGTAAAGAAGTAAAAGCTCGTATCCCATTTCCATTCCCTCCTG
AAACAGCAACTTGTCGTGTATCTGTTGCTCCATTACCATCTATCACATATGAAGAATCAGAC
GTACTATTAGCTGGTACTGCTAAATACCCTGTATTATTAACAACACGTAACCTTGGTTTCCA
CTCTAACGCAACTTCTGAATGGATTCAAGGTAAATATTTACGTCGTATTCCAGTTACTCCTC
AAGGTATTGAATTAACTTGGGGTAACAACGCTCCTTTACACTTCTGGTCATCAGTACGTTAT
GCTTCAGGTGATGCAGATGCTTATCCATGGAATTATTAGTTCATCACACAAAACACCACGG
TGGTGGTGGTTCTTACCCATACGATGTTCCTGACTACGCTTAAtaattttatttttcatga
tgtttatgtgaatagcataaacatcgtttttatttttatggtgtttaggttaaatacctaaa
catcattttacatttttaaaattaagttctaaagttatcttttgtttaaatttgcctgtctt
tataaattacgatgtgccagaaaataaaatcttagcttttttattatagaatttatctttat
gtattatattttataagttataataaaagaaatagtaacatactaaagcggatgta

Key

- C. reinhardtii promoter and UTR sequences
- Cholera toxin beta
- Flexible GGGGS linker units
- Residues 353-729 from the structural polyprotein of SAV subtype 3 (Genbank Acc. No. AGTT2234)
- HA epitope tag

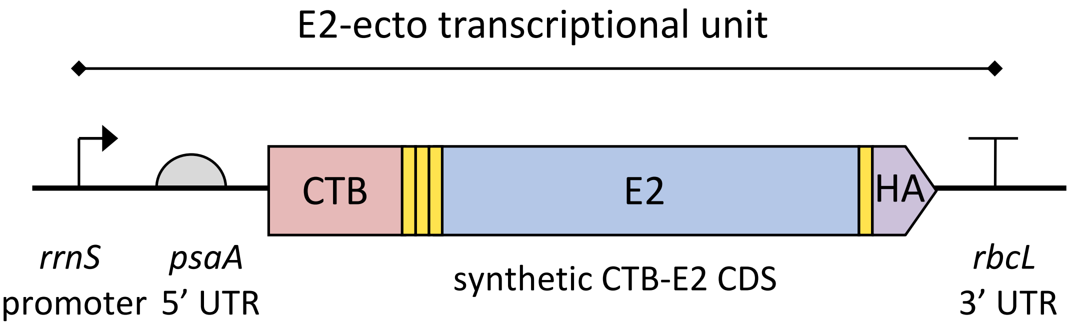

>Protein sequence of E2-ecto

```
MTPQNITDLCAEYHNTQIYTLNDKIFSYTESLAGKREMAIITFKNGAIFQVEVPGSQHIDSQ
KKAIERMKDTLRIAYLTEAKVEKLCVWNNKTPHAIAAISMANGGGGSGGGGSGGGGSAVSAS
PAAVYDTQILAAHAAASPYRAYCPDCDGTACISPIAIDEVVSSGSDHVLRIRVGSQSGVTAK
GGAAGETSLRYLGRDGKVHAADNTRLVVRTTAKCDVLQATGHYILASCPEGQSITVAATLDG
TRHQCTTVFEHQVTEKFTRERSKGHHLSDLTKKCTRFSTTPKKSAPYLVDVYDALPISVEIS
TVVTCNDNQCTVRVSPGTTVKFDKKCKSAAQATVTFTSDSQTFTCEEPVLTAASITQGKPHL
RSSMLPSGGKEVKARIPFPFPPETATCRVSVAPLPSITYEESDVLLAGTAKYPVLLTTRNLG
FHSNATSEWIQGKYLRRIPVTPQGIELTWGNNAPLHFWSSVRYASGDADAYPWELLVHHTKH
HGGGGSYPYDVPDYA
```
